# Epidemiology and Transmission of Kaposi’s Sarcoma-Associated Herpesvirus

**DOI:** 10.3390/v6114178

**Published:** 2014-11-04

**Authors:** Veenu Minhas, Charles Wood

**Affiliations:** Nebraska Center for Virology, School of Biological Sciences, University of Nebraska, Morrison Center, 4240 Fair Street, Lincoln, NE 68583, USA; E-Mail: vminhas2@unl.edu

**Keywords:** KSHV, HHV-8, epidemiology, transmission

## Abstract

This review summarizes the current knowledge pertaining to Kaposi sarcoma-associated herpesvirus (KSHV) epidemiology and transmission. Since the identification of KSHV twenty years ago, it is now known to be associated with Kaposi’s sarcoma (KS), primary effusion lymphoma, and multicentric Castleman’s disease. Many studies have been conducted to understand its epidemiology and pathogenesis and their results clearly show that the worldwide distribution of KSHV is uneven. Some geographical areas, such as sub-Saharan Africa, the Mediterranean region and the Xinjiang region of China, are endemic areas, but Western Europe and United States have a low prevalence in the general population. This makes it imperative to understand the risk factors associated with acquisition of infection. KSHV can be transmitted via sexual contact and non-sexual routes, such as transfusion of contaminated blood and tissues transplants, or via saliva contact. There is now a general consensus that salivary transmission is the main route of transmission, especially in children residing in endemic areas. Therefore, there is a need to better understand the sources of transmission to young children. Additionally, lack of animal models to study transmission, gold standard serological assay and the lack of emphasis on endemic KS research has hampered the efforts to further delineate KSHV transmission in order to design effective prevention strategies.

## 1. Introduction

Human herpesviruses are large double stranded DNA viruses that are ubiquitous in nature. Of the eight known human herpesviruses two have been implicated to be the etiologic agents for a number of cancers, Epstein-Barr virus, and the Kaposi sarcoma-associated herpesvirus (KSHV). Since its identification in 1994, KSHV is known to be associated with all forms of Kaposi’s sarcoma (KS) primary effusion lymphoma and multicentric Castleman’s disease [1,2,3,4]. KS is an AIDS-defining illness and is the most common malignancy present in HIV-1 infected patients [5]. One of the earliest indicators of the upcoming HIV/AIDS epidemic was the sudden appearance of KS and high-grade non-Hodgkin’s lymphoma in young men having sex with men (MSM) [6].

Since KSHV DNA cannot be detected in all infected individuals, a serological assay to detect the presence of antibodies against KSHV is the method of choice to investigate KSHV prevalence in a population. Unlike EBV, global seroprevalence of KSHV varies greatly and is generally high in areas where non-HIV associated forms of KS (classic or endemic forms) have been common [7]. These areas include African and Mediterranean regions, where KSHV seroprevalence ranges from 20% to 80% in the adult populations, whereas seroprevalence in the United States and Northern Europe is generally low (<10%) [8,9,10,11,12,13,14]. In South America, the Amerindians have also been identified as a hyperendemic population for KSHV infection [15]. Interestingly, the prevalence in the non-Amerindians is significantly lower. de Souza *et al.* have recently reported that >70% of children (four to nine years) were positive for KSHV and the prevalence increased with age (>90% in >40 year age group) [16]. This pattern is strikingly similar to Africa, where infection seems to occur in early childhood. In contrast, KS incidence is uncommon and KSHV prevalence is low in most Asian countries [17]. However, in China, KSHV prevalence varies considerably between different regions of the country and is between 7.3% percent and 16.1 percent in adults in different provinces [18,19,20,21]. In particular, the prevalence was found to be high in the Xinjiang province, which is located in the northwestern region of China. Interestingly, cases of both classic KS and AIDS-associated KS are found mostly in the Uyghurs and the Kazakh ethnicities [20,21]. The reasons for such geographical and population variations of KS and KSHV distribution remain to be addressed.

Our laboratory has conducted extensive cohort studies in Zambia, a sub-Saharan African country. Zambia has been considered as part of the “KS belt”, where endemic KS was prevalent and where significant increase in KS incidence in adults and children has coincided with the emergence of the HIV-1 epidemic [22,23,24]. This increase in KS incidence is significant because, by 1992, KS accounted for approximately 25% of all childhood cancers diagnosed in Lusaka, the capital of Zambia [25]. This review summarizes some of the results of our cohort studies in Zambia, together with those from other significant studies that have contributed to further our understanding of KSHV transmission and pathogenesis, and, more importantly, explore some of the questions that still remain unanswered and warrant further research.

## 2. Modes of Transmission

Primary KSHV infection can occur during childhood and as an adult and can be transmitted via both sexual and non-sexual routes. KSHV can be found in the peripheral blood mononuclear cells (PBMCs), saliva, oropharyngeal mucosa, semen, cervico-vaginal secretions, and prostate glands, which may represent the source of both vertical and horizontal transmission [26,27,28,29]. The modes of transmission of KSHV may vary in different parts of the world, depending on the endemicity of that region. In non-endemic areas, especially in adults, sexual transmission (homosexual and heterosexual) of KSHV may be the route of transmission [30,31,32]. However, there is limited information about this route and further extensive studies are needed. Studies have shown that sexual transmission, particularly among MSMs, may play a major role in transmission in non-endemic areas, such as the United States and Western Europe, because the seroprevalence among MSMs is significantly higher as compared to the general population. In the San Francisco Mens Health Study, the prevalence of KSHV infection was reported to be high among MSMs (37.6%) when compared to heterosexual men (no prevalence in this group) [31]. They also reported an association of KSHV prevalence with the number of sexual partners. Other studies have reported that besides the number of sex partners, syphilis infection and hepatitis B infection are also risk factors for acquisition of infection [27,31,33,34]. However, the possibility of sexual transmission among heterosexual individuals remains unclear. Different studies have been inconsistent about the evidence of transmission through heterosexual contact. This can be explained in part by the populations that were investigated. Two African studies have been conducted in commercial sex workers in Nigeria and Kenya, and clearly suggest sexual transmission occurring in the adult population [35,36]. Another study conducted in truckers in Kenya also suggest that there is ongoing sexual transmission during adulthood [37]. However, there are other studies that do not observe any evidence of sexual transmission [38,39]. One of them is a study conducted by Malope *et al.*, in South Africa. This is an interesting study because it had a large sample size and sampling was conducted from miners, sex workers, and other residents of the city. This study did not find any association of KSHV prevalence with sexual behavior. In addition, our study conducted in a group of female sex workers in China showed KSHV prevalence of 10%, which is similar to the general women population, even though the prevalence of other sexually transmitted infections (STI), such as HSV-2 and syphilis, were higher than the general women population. Our study suggested that heterosexual transmission may not be a dominant route for KSHV, at least among Chinese women [40]. Another important issue while investigating sexual transmission is that both kissing and salivary exchange during hetero and homosexual contact is common. This makes it harder to delineate whether the transmission was sexual or oral through saliva. Additionally, it is important to consider that all these studies were cross-sectional in design. Longitudinal cohort studies that can follow individuals for seroconversion and incident infection are needed to truly understand the impact of this route of transmission.

Besides sexual transmission, another possible horizontal transmission route is via KSHV contaminated blood transfusion because viral DNA may be detected in 10–15 percent of PBMCs of healthy KSHV seropositive individuals [41,42]. Transmission of KSHV via this route is likely to be inefficient because of the cell associated nature of the virus, and viremia is uncommon, even in KSHV infected individuals. However, blood transfusion has recently been investigated in an elegant study by Hladik *et al.*, where they provided strong evidence that KSHV can be transmitted by blood transfusion [43]. This study was conducted in Uganda, where 991 KSHV seronegative recipients were followed after transfusion. Among them, 425 patients received KSHV seropositive blood and 566 patients received KSHV seronegative blood. They observed that KSHV seroconversion occurred in 41 of the 991 recipients. The excess risk of KSHV seroconversion after transfusion with KSHV seropositive blood (during the observed 24-week follow-up) was 2.8%. This suggests that 12 patients (of the 425 patients exposed to KSHV seropositive blood) were infected by transfusion. Another interesting result of this study was that an excess risk of 4.2% was observed among patients who received blood stored for less than four days as compared to those who received blood stored for more than four days. The results from this study led them to further investigate whether transfusion of KSHV positive blood led to an increase in mortality. Their results show that transfusion of short stored blood was associated with increased risk of death (adjusted hazard ratio – 1.79) [44]. Whether the increased risk was due to KSHV or other factors in short stored blood will need to be further investigated.

After KSHV was identified, several observations and published studies led researchers to hypothesize that KSHV can be transmitted by another non-sexual route, specifically from mother to child vertically. The high seroprevalence level in children, especially in endemic areas was one of the rationales. Additionally, KSHV has been found in cervico-vaginal secretions of HIV-1 and KSHV co‑infected women, suggesting that KSHV viral load in the female genital tract might influence the vertical transmission of KSHV [45]. Moreover, development of KS in children less than one year of age was indicative of perinatal transmission [46]. These observations also formed the basis of our cohort study conducted between 1998 and 2004. Pregnant mothers in early stages of labor were recruited in the cohort and both the mother and infant were enrolled and followed after delivery. Vertical transmission was investigated in 89 mother-infant pairs. All of these 89 children were born to KSHV seropositive mothers whose serostatus was tested at the time of delivery [28]. We have reported that KSHV DNA was detected in PBMCs in 2/89 children within 24 h after birth. More recently, Lisco *et al.* have addressed this question from a different perspective [45]. As herpesvirus reactivation occurs during pregnancy, they investigated the KSHV presence and viral load in PBMCs and cervicovaginal secretions (CVS) from 15 pregnant Italian women. They have reported viral reactivation in 5/15 women in PBMCs and CVS. One infant also had the same viral subtype as the mother at two months after birth. Together, these reports strongly indicate that *in utero* or intrapartum KSHV infection might, albeit rarely, occur in countries where KSHV is endemic. More studies, with larger sample sizes are required to further delineate this route of transmission.

Breast milk transmission has also been hypothesized to be contributing to KSHV transmission from mother to infants. Breast milk has been reported to contain herpesviruses, such as CMV, EBV, and HHV-7 [47,48,49,50,51]. Both CMV and KSHV are known to infect monocytes, macrophages, and epithelial cells. These cells are found in the cellular components of mature milk suggesting that KSHV might also be found in breast milk. Indeed, a study by Dedicoat *et al.* has shown that KSHV DNA could be detected in 12/43 breast milk samples from South African mothers [52]. Our laboratory has investigated the presence of KSHV DNA both in breast milk cells and in the liquid portion of breast milk (supernatant) [53]. However, we failed to detect any viral DNA in breast milk, whereas EBV DNA was readily detectable. Since then, there has been no recent study that has investigated the potential of breast milk transmission. Therefore, the current evidence does not point towards breast milk transmission being an important route of horizontal KSHV transmission from the mothers to their infants, at least in Zambia.

The high prevalence of KSHV infection reported by many studies, especially those conducted in African children cannot be explained by transmission routes discussed above; vertical mother to child transmission, breast milk transmission or via blood contamination. Therefore, another major horizontal route of transmission that needs to be investigated is salivary transmission. Indeed, several interesting studies indicated that saliva as the likely candidate, which mediates childhood transmission. Soon after its discovery, there were studies that reported the presence of KSHV DNA sequences in saliva [54,55,56]. In 2000, Pauk *et al.* reported that oral exposure to infectious saliva was a potential risk factor for the acquisition of KSHV among men who have sex with men. Since then, our group and others have conducted several studies to show that KSHV is shed in saliva of infected individuals regardless of their HIV-1 status [53,57]. We have reported that the group of mothers who were not shedding KSHV in breast milk, did shed KSHV in saliva (19/65 samples were positive). Other studies have also investigated the frequency of shedding over a period [57,58]. The results showed that not all KSHV seropositive individuals shed the virus in saliva, and individuals in whom KSHV can be detected can range from occasional shedders to daily shedders. All the above studies provided strong evidence that horizontal transmission, predominantly via saliva, is the major route of transmission especially in endemic countries.

## 3. Sources and Risk Factors Associated with Transmission

Soon after the discovery of KSHV, a majority of the studies conducted in Africa, even though cross-sectional in design, indicated that KSHV infection was prevalent in young children [11,59,60]. Our cohort studies first started in 1998 with the aim to study the epidemiology of KSHV in Zambia. Our group has reported that more than 10 percent of Zambian children may be infected by 12 months of age [61]. Other studies, though cross-sectional in design, have also shown that especially in endemic regions, primary infection likely occurs in early childhood leading to accumulation of infection. This is evident from the observed high seroprevalence during childhood (Table 1). Therefore, it is important to understand the source of the transmission of virus to children and the risk factors associated with acquisition of infection. The exact risk factors that predispose children to acquisition of KSHV infection are still not fully understood but immunosuppression, especially due to HIV-1 infection is a major risk factor. The route(s) of transmission most likely varies among populations, and given the increasing availability of anti-retroviral therapy (ART) in the high HIV-1 prevalence regions, such as sub-Saharan Africa, there is a need to study the effect of ART on transmission to HIV-1 infected individuals including children.

It has been reported that there is an increased prevalence of KSHV antibodies in children of mothers shedding high number of viral DNA copies/ml of saliva, suggesting that KSHV in maternal saliva may be associated with transmission to the child [62]. As mentioned above, we have observed KSHV shedding in maternal saliva. Therefore, it will be important to determine whether the maternal KSHV shedding in saliva is important for transmission to children and the specific common practices that are associated with transmission of infection. The specific practices that expose individuals, especially young children, to salivary transmission still remain to be explored and are a subject of our ongoing research study in Zambia. Other studies also indicate that, in Africa, transmission from mother to child and between siblings accounts for a substantial proportion of childhood infections [29,60]. Preliminary analysis of our ongoing cohort studies in Zambia reveal that transmission to children also occurs in households where no household member is KSHV seropositive, who could serve as a source of infection to the child. This indicates that transmission may occur from sources outside the family unit. In fact, we found that children from several families have KSHV genotype that differed from those of their mothers and other family members, suggesting that they have acquired KSHV infection from sources outside the household [63]. Other than these studies from Africa, very little is known about transmission of KSHV in children in developed countries, including the United States, since the prevalence of childhood infection is low, and the results of reports from these countries are conflicting [11].

**Table 1:** Selected recent reports summarizing the observed percent seroprevalence of KSHV in early childhood.

Reference (Year)	Country	Age (Years)	Percent Prevalence
Cao *et al*. (2014) [21]	Xinjiang, China	0.5–5	48.3
Wakeham *et al*. (2013) [64]	Entebbe, Uganda	1	4
		2	7
		3	10
		4	13
		5	14
Butler *et al*. (2011) [65]	Buziika B Parish, Uganda	1.5–2	15.5
		3–5	22.7
		6–9	31.6
		10–13	32.0
Pfeiffer *et al*. (2010) [66]	Kampala, Uganda	0–4	32.1
		5–9	37.4
		10–19	30.6
	Shirati District, Tanzania	0–4	33.9
		5–9	33.7
		10–19	32.4
	Lagos, Nigeria	0–4	14.6
		5–9	2.3
		10–19	83.1
Butler *et al*. (2009) [38]	Kampala, Uganda	2–8	10–30.6

**Figure 1:**
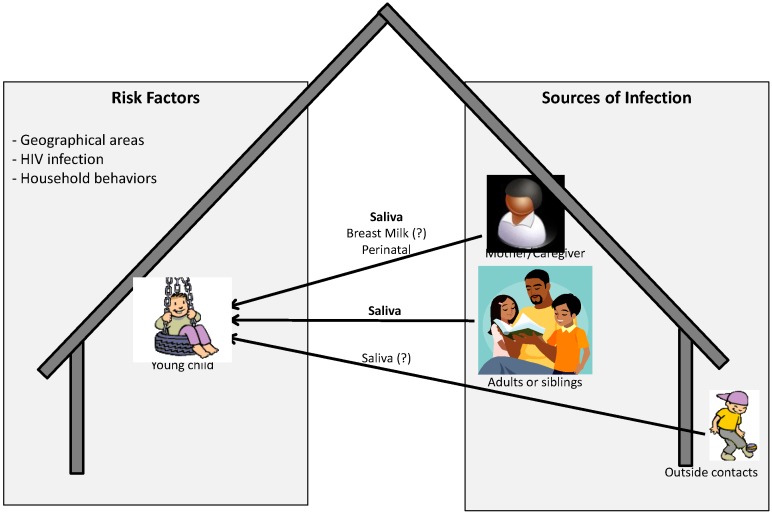
Pictorial representation of possible sources and risk factors associated with acquisition of KSHV infection by children in endemic areas.

The current knowledge about source and route of infection, and risk factors associated with acquisition of infection has been summarized in Figure 1. As described in detail above, for young children, especially in endemic areas where children can be infected by members from within and outside the family mainly via saliva, other routes are possible but may not be the major routes of transmission. In addition, factors present both within and outside the household or in the environment may also increase the risk of acquisition of infection by the child. In fact it has been reported that environmental or ecological factors may be associated with the development of KS. This includes geographical areas with volcanic soils, chronic schistosome or other parasite infections, biting flies or contact with phorbol esters or other constituents of plants [67,68,69,70]. Whether these factors also increase the risk of acquisition of KSHV infection is not known.

## 4. Future Research

The year 2014, marks the 20^th^ year since the discovery of KSHV. This is a time to reflect on the great strides achieved towards understanding the biology and epidemiology of KSHV, and look towards the future to explore other critical questions that need to be investigated. KSHV pathogenesis is an area of active research and animal models have been used to study several aspects of KSHV infection and pathogenesis. However, there was no ideal model available to understand KSHV transmission, routes of transmission, early events following primary viral infection and its interaction with the host particularly regarding disease development. One of the main hurdles was the lack of an effective model that supported natural routes of viral infection followed by a long-term sustainable infection involving both latent and lytic viral gene expression, and leading to the infection of target cells and tissues. There have been several studies that have developed animal models to study KSHV infection; they include the non-human primate model. However, primate models are difficult to work with and not all reported models were able to establish persistent infection [71,72]. Therefore, there is still a great need to develop small animal models, such as a rodent model, which can be used to study KSHV infection and pathogenesis efficiently. Such a model will need to have relatively short generation time, inexpensive, and easy to manipulate. Additionally, this model needs to support natural route of viral infection, a long term sustainable infection involving both latent and lytic viral gene expression, and the infection of target tissues and cells that reflect those of human infection. Recently, a new generation of humanized mouse, the BLT (bone marrow, liver and thymus) mouse (hu-BLT) has been shown to be an excellent model for studying human viral infections [73]. This is the only mouse model which can generate the human mucosal immune systems, and a human HLA restricted antigen specific humoral and cellular responses [74]. To this effect, our laboratory has recently used this mouse model to study KSHV infection [75]. Our study demonstrated that both latent and lytic viral transcripts, as well as viral protein expression in various tissues, including spleen and skin. Interestingly, mice could be infected via several routes, including the oral mucosal route. Furthermore, we found that KSHV can establish infection in human B cells and macrophages in this model. Our study represents the first successful effort to recapitulate KSHV infection in a small animal model via a natural route of infection. The hu-BLT mouse could potentially be further developed as a model not only for studying the pathogenesis of KSHV *in vivo*, but can also be applied to study routes and mechanisms of KSHV transmission and systemic dissemination.

As discussed earlier by a number of reports, there is still a lack of a gold standard serological assay that is equally effective in detecting anti-KSHV antibodies in KS patients (generally having high antibody titers) and in KSHV infected but non-KS individuals (generally having lower antibody titers). Lack of such an assay has made it difficult to develop a clearer global picture about KSHV epidemiology; since the performance of various assays differs, and it has been difficult to compare across studies that also differ in the characteristics of the population and study design. Currently, there now are two general platforms of KSHV serologic assays, the ELISA based assay and the immunofluorescence-based assay. The ELISA based assays in general involve the use of recombinant KSHV structural proteins. Such an assay is objective and of high throughout, readily screening through a larger number of samples. However, the common format involves the use of only two or three viral proteins, and it is known to underestimate the prevalence of infection in a study population. In addition, setting the cutoff is critical because the titers of KSHV antibodies vary greatly between different study populations and between geographical locations. Recently, Labo *et al.* have reported an important study about the development of a bead-based multiplex assay that detects antibodies to six KSHV antigens [76]. Interestingly, the authors conducted a systematic antigenic analysis of the entire KSHV proteome by screening 72 KSHV proteins to understand the seroreactivity pattern. Further evaluation of this assay on a larger sample size composed of different study populations may prove this approach to be very useful for KSHV serodiagnosis.

The second KSHV serology platform is immunofluorescence assay (IFA). This assay in general utilizes KSHV chronically infected B lymphoma cell lines such BC3 cells, which are latently infected with KSHV. These cells are then stimulated by TPA to undergo lytic reactivation so that both lytic and latent viral proteins can be expressed effective, and then used for the detection of anti-KSHV antibodies in study population using indirect immunofluorescence [77]. There are a number of laboratories, including ours, that have used this assay format because of the sensitivity of the assay and its ability to detect all viral antigens expressed. Our laboratory has used this assay to conduct a number epidemiology studies in different study populations, both in Zambia and China [40,61]. However, such an assay is subjective, labor intensive, has low throughput and also required highly experienced laboratory personnel, and IFA is not a high throughput assay when compared to ELISAs. Using such assays in Zambia, we have observed that children generally have lower titers as compared to adults. Development of a more efficient highly sensitive and standardized assay will be important to further confirm the seroprevalence of KSHV in different parts of world, especially in non-KS individuals. Use of such an assay will also help to confirm whether the currently reported seroreversion in infected individuals could be due to a true drop in antibody titers to below detection limit of our current assays, or whether there is a true loss of KSHV antibody with time due to the viral latency [41,61].

KSHV antibodies, for a large part, has been used to investigate KSHV epidemiology, but there is still a need to understand the role of the immune response in preventing KSHV infection, in controlling viral infection and the development of the KS in infected individuals. There is also a need to further study not only the role of the humoral immune response but the cellular immune response as well. For the humoral response, an in-depth understanding of the role broadly protective immune response on the control of KSHV infection and development of KS will be important. Two studies have quantified the prevalence and titers of neutralizing antibodies (nAb) in KS patients or in KSHV infected asymptomatic controls. Both reports have focused on a small number of KS patients from the US. One suggested that KS patients had lower titers of neutralizing antibodies compared to asymptomatic individuals irrespective of their HIV status [78], while the other study found no significant difference between the two groups [79]. In addition, the overall prevalence of nAb in KS patients or asymptomatic subjects was found to be low and comparable between KS and asymptomatic individuals [79]. Our laboratory has investigated the prevalence and titer of KSHV nAb in a cross-sectional cohort of Zambian patients [80]. HIV-1-associated KS patients had a significantly higher prevalence of neutralizing antibody response when compared to non-KS but KSHV infected individuals. This suggested that viral lytic replication and the resulting antigenemia, which occur during KS development, might be essential contributors to development of neutralizing responses. However, whether these responses are protective if passively introduced prior to primary KSHV infection is currently not known. In addition to nAb, further studies are required to decipher the role of cell-mediated immunity in protecting against KSHV infection and preventing the development of KS. A major hurdle in this area of research has been a lack of a robust T cell response against KSHV antigens. A weak adaptive immune response following primary infection has proved to be the major obstacle in this area. However, recent studies have provided some interesting data. One of these studies by Lepone *et al.* is very interesting that has reported on the identification of five novel HLA A*0201-restricted CD8^+^ T cell epitopes present in LANA-1, K12, gB, and K8.1 proteins [81]. These epitopes induced both single and multiple immune mediators in CD8^+^ T cells from healthy KSHV seropositive individuals. A review by Robey *et al.* has provided a list of identified epitopes to known KSHV immunogenic open reading frames [82]. These are critical steps towards understanding the role of T cells in the pathogenesis of KSHV infection though further research in identification of other immunodominant epitopes is needed. Further studies are also required to understand the role of CD8+ and CD4+ T cell responses to primary infection and disease development. There is a gap in our understanding of broad range of antigenic epitopes that can be restricted by other MHC class I and II haplotypes.

In addition to the immune response against KSHV, there needs to be a better understanding of the routes of transmission in different populations, the risk factors associated with incident infection and the potential source(s) of transmission. This information is pertinent in the public health disease management for the design of strategies to prevent KSHV infection, especially in endemic regions. Prevention of transmission to high-risk individuals in Western countries and to children in endemic areas, especially in Africa, may be the best approach to reduce the burden of infection and the subsequent development of KS. We believe that further work in understanding local risk factors and sources of transmission will lead to the development of strategies to prevent transmission of infection to susceptible individuals.

In parallel to the development of public health policy to prevent KSHV infection, there is also a need towards the development of protective vaccines since vaccination is the most efficient method to eliminating viral infectious agents and to curb the epidemic. However, there are ongoing discussions among researchers and policy makers whether such a vaccine is needed for KSHV since the KS incidence is decreasing because of the effectiveness of the ART, at least in the developed countries [83,84]. However, due to the high prevalence of KSHV in the African countries and in other KS endemic regions, it is questionable whether ART and other public health prevention strategies will be effective in preventing KSHV infection and the subsequent risk in developing KS. Therefore, there is a still a continuous need to better understand the viral antigens and the generation of protective immune response against KSHV antigens. Such an understanding will be a first step towards developing a protective KSHV vaccine.

In addition to the need to prevent epidemic KS due to its sudden rise in concordance to the AIDS epidemic, there is a severe lack of information about the endemic KS. This form of KS is still prevalent in many parts of Africa and its epidemiology and pathogenesis needs to be better characterized. It is most likely that the endemic KS will continue to persist in these regions due to the high prevalence of KSHV infection, even in the HIV-1 uninfected population. We have recently conducted a study at the Dermatology and Venereology clinic at University Teaching Hospital, Lusaka, Zambia, which is a national referral clinic for all suspected KS cases. From 2008 to 2013, we document 726 pathologist confirmed KS cases at this clinic [85]. Of these, one third of cases were diagnosed in HIV negative patients (endemic KS). This underscores the need to understand the epidemiology of endemic form of KS in this region. Currently, there is a gap in the literature regarding the incidence of endemic KS, whether prevalence is stable or increasing, risk factors associated with development of endemic form of KS. Up to now endemic KS has received little attention because it is overshadowed by the recent epidemic KS, especially when a number of studies have reported a decrease in epidemic KS incidence in anti-retroviral treated HIV positive patients, even though they are mostly occurring in the developed countries [83,85,86]. Our study in Zambia clearly shows that the endemic KS is still prevalent and it is likely that antiretroviral therapy will have little or no impact on its incidence. Therefore, it is likely that endemic areas will continue to bear the burden of endemic KS. Furthermore, while great strides have been taken in increasing the coverage of ART in HIV-1 positive patients, the degree of coverage in different African countries varies. Therefore, to further reduce epidemic KS in areas that are endemic for KS (such as the “KS belt” in Africa) without complete ART coverage may even prove difficult in the near future. Our study in Zambia and other studies have clearly shown that classic and endemic forms of KS are more prevalent in males as compared to females [85,87]. These observations also raise the question that genetic factors linked to gender may have a role in progression to KS.

While conducting our cohort studies in Zambia, we have observed that laboratory research findings have yet to be translated into public health practices and programs to reduce transmission of KSHV. Health care professionals are often not aware of KSHV and its link to KS, and current research in this field and better programs similar to human papilloma virus, hepatitis B virus and HIV-1 prevention need to be developed. Educational programs focusing on prevention of transmission are especially needed in endemic regions with limited resources for treatment of KS. It is imperative that results of laboratory research be translated into effective health behavior interventions for prevention of KSHV infection and for overall community health promotion.
